# Improving knowledge about disability transitions by adding retrospective information to panel surveys. Population Health Metrics

**DOI:** 10.1186/1478-7954-4-16

**Published:** 2006-12-13

**Authors:** James N Laditka, Douglas A Wolf

**Affiliations:** 1 Department of Epidemiology and Biostatistics, Arnold School of Public Health, University of South Carolina, 800 Sumter Street, Columbia, South Carolina 29208, USA; 2 Center for Policy Research, Syracuse University, 426 Eggers Hall, Syracuse University, Syracuse, NY 13244-1020, USA

## Abstract

**Background:**

Panel data are often used to estimate key measures of public health, such as years lived with and without disability. Panel surveys commonly measure disability at intervals of one or two years, and occasionally more than two. It is likely that these intervals often include unreported changes in functional status. Unreported changes may bias estimates of disability transition probabilities, which are commonly used to estimate years lived with and without disability. Most surveys do not ask participants about periods with and without disability in the time since they last responded to the survey. We examined a way to improve the usefulness of panel surveys and our understanding of disability processes, by eliciting retrospective disability information.

**Methods:**

Data were from the United States' National Long Term Care Survey. At each wave, this survey asks disabled respondents how long they have been disabled. We tested whether estimates of probabilities predicting changes in disability status can be improved by making use of this retrospective disability information. Methods included embedded Markov Chain analysis, microsimulation, and the Hausman specification test.

**Results:**

Estimates based on data that include retrospective information are significantly different from those that use only the more limited information that is contemporaneous to the surveys. They are also more efficient. At age 65, all estimated probabilities for becoming disabled were higher when retrospective information was used, and all probabilities for remaining disabled were lower. Microsimulation revealed that using retrospective information increased the number of functional status transitions. For example, for women the mean number of transitions from nondisabled to disabled or dead was 52.7% greater when retrospective information was added to the analysis.

**Conclusion:**

Our results suggest that the value of future panel studies for estimating transitions in disability could be notably enhanced by adding a small number of questions asking respondents for details about their disabilities–and *lack *of disabilities–in the period since a preceding survey wave. Information provided by such questions could substantially improve both the measurement of disability histories and estimates of disability processes.

## Background

There are commonly one or more years between survey dates in the longitudinal studies used in disability research. Although the literature includes reports of health transitions recorded over time intervals as long as 19 years [1], it is unusual to find estimates of disablement-process parameters–i.e., transition rates or transition probabilities between health or disability statuses–based on data that span more than a two-year period. Data from the Massachusetts Health Care Panel Study and the Americans' Changing Lives study have been used to estimate 10-year and 8-year transition probabilities, respectively [2,3]. Data from the five-year intervals used in the National Long Term Care Survey (NLTCS)–the data source used in this paper–have been used to estimate one-year transition probabilities [4] as well as monthly rates of change in functional states [5]. More common are estimates based on two-year measurement intervals such as those found in the Retirement History Survey [6], the Longitudinal Study of Aging [7] or the Health and Retirement Survey [8].

It is likely that many individuals experience changes in functional status during the long intervals that often separate survey waves. Information about these changes is not usually captured by the surveys. There are two ways to address the problem of undetected disability transitions in panel data. One way is to survey respondents more frequently. This approach is illustrated by a study that assessed respondents' disability status every month for 53 months [9]. Results suggested that a large proportion of older individuals experience episodes of disability, and that many of these individuals recover quickly. This relatively frequent turnover in disability status highlights a limitation of relying on panel data with long intervals between survey waves. In practice, however, limited funding and concerns about respondent burden make it difficult to conduct more frequent surveys of the same individuals, especially in widely-dispersed national samples.

Alternatively, follow-up interviews could include questions about periods with and without disability in the time since the preceding survey wave. Although this approach is likely to involve more measurement error, it can be implemented in existing panel survey designs with little additional cost or respondent burden.

We explore the second approach, employing what appear to be previously unused data elements included in the NLTCS. For each of six activities of daily living (ADLs), the NLTCS includes questions about the *duration *of any existing disability. If respondents say they are disabled in an ADL, they are asked how long they have been disabled. Responses are interval-coded, producing uncertainty regarding the exact time when a current disability began. Nonetheless, for some respondents we can substantially increase available information about disability spells by using the duration variables.

Our approach is similar in spirit to that of Yi, Danan, and Land [10], who augmented conventional panel-survey data on disability with information on the disability status of decedents immediately prior to death. Thus otherwise unrecorded information on the *presence *(but not the *duration*) of disability was used to adjust estimates of active life expectancy. The adjustment showed that conventional measures of active life and inactive life expectancy were biased by as much as 15% in some age and gender groups.

We use the embedded Markov chain (EMC) approach to the problem of missing data presented by unrecorded functional status in the periods between survey waves. The EMC expresses the probability of an observed sequence of functional status in terms of single-period transition probabilities, and uses maximum likelihood techniques to estimate parameters representing the single-period transitions. In our research, the single-period transition probability is estimated for each month. Laditka and Wolf [11] introduced EMC methods for research on Active Life Expectancy, which estimates the length of time an individual can expect to live both with and without disability. The method has become widely used in Active Life Expectancy research [12-15], and in related research on health processes [16,17].

## Methods

### Data characteristics and research datasets

We analyze respondents' reports of disabilities in six ADLs, using the 1982, 1984, 1989 and 1994 survey waves of the NLTCS. In 1982, screening interviews were administered to a sample of Medicare beneficiaries aged 65 and older. Non-institutionalized individuals who were identified through screening as having a chronic disability in any ADL received detailed follow-up interviews. In the NLTCS survey design, chronic disability was defined as an inability to perform any ADL without the aid of another person or equipment that had lasted, or was expected to last, at least 90 days [4]. Chronic disability was required for an individual to "screen in," to receive a detailed interview. In later waves, those who did not receive a detailed interview in a previous wave were screened again. They also received follow-up interviews if they reported chronic disability. Individuals with detailed interviews in any survey wave were automatically given the detailed interview in subsequent waves. NLTCS sampling information has been published [4,18].

The screening interviews provide only limited retrospective information on the duration of disability. Those reporting disability in an ADL were asked if it had existed for "at least three months." Respondents to detailed community interviews were asked if the disability had existed "less than 3 months," "3 months to less than 6 months," "6 months to less than 1 year," "1 year to less than 5 years," or "5 years or over." Duration questions were asked separately for each ADL.

From the full NLTCS sample, we initially selected a sample of all individuals who participated in the 1982 survey at ages 65–69 (*n *= 5523). We excluded institutionalized individuals (*n *= 144), because their 1982 survey did not elicit specific ADL information. We created longitudinal arrays representing each of the six ADLs: eating, dressing, getting in or out of bed, getting around inside ("mobility"), dressing, bathing, and getting to the bathroom or using the toilet ("toileting"). Each cell in each array represented a single month. The leftmost cell in each array corresponds to the month of the individual's 65^th ^birthday. We then used information from screening interviews, community interviews, and institutional interviews to assign ADL disability status, either disabled or nondisabled, to the array cells corresponding to the months in which the information was obtained. We defined disability as use of help or assistive devices. Because we focus on functional status *transitions*, we imposed the further requirement that there must be at least two months of non-missing status information in each of the six ADL arrays, or at least one month of ADL status and the month of death. This requirement reduced the sample to *n *= 5360.

Analysis of longitudinal survey participation revealed that 1909 individuals in our sample were not included in the 1989 survey, despite their presence in earlier waves and in the 1994 survey. These individuals were excluded in 1989 due to limited funding [19]. Because of the long interval between interviews, we excluded these respondents from the analysis. This reduced the sample to *n *= 3451. Finally, we chose as the right-censoring point the latest month in which all six arrays contained non-missing status information. This requirement removed 11 observations with incomplete information. The final analytic sample had 3440 observations. Demographic characteristics of the initial sample, and of the final analytic sample, appear in Table 1. About 53.5% of the final sample was female. About 9.8% was nonwhite.

**Table 1:** Demographic Characteristics of Initial Non-Institutionalized Sample Age 65–69 in 1982 (Sample 1, *n= *5379)^a ^and Final Sample (Sample 2, *n *= 3440). National Long-Term Care Survey, Percent Distribution

	Men				Women				Total	
			
	White		Non-White		White		Non-White			
Sample:	1	2	1	2	1	2	1	2	1	2

1982 Age										
65	5.9	5.8	0.5	0.4	6.9	5.5	0.7	0.6	14.0	12.4
66	8.9	8.5	0.8	0.7	11.5	9.6	1.1	1.1	22.2	19.9
67	8.8	8.9	0.8	0.9	11.1	9.7	1.2	1.2	22.0	20.6
68	8.4	8.3	0.8	0.8	11.2	9.6	1.2	1.3	21.6	20.1
69	8.0	10.9	0.8	1.2	10.3	13.4	1.1	1.5	20.2	27.0
Total	40.0	42.4	3.6	4.0	51.1	47.8	5.3	5.8	100	100
Male									43.7	46.5
Female									56.3	53.5
White									91.1	90.2
Non-White									8.9	9.8

For waves in which an individual participated in both the screening interview and a community or institutional detailed interview, the arrays capture his or her status in each of the ADLs in both occasions. Because we did not impose the condition that respondents must have been, or expect themselves to be, restricted for at least 90 days in order to be coded as disabled, months representing screening interviews include ADL disability that may not have met the NLTCS definition of chronic disability.

The arrays also capture information about disability *duration*. In instances where a respondent to a screening interview indicated that an ADL disability had not lasted at least 3 months, we inferred that the disability existed in the month prior to screening, but not before. We assigned a disability code to the corresponding array location. We also knew from such responses that, subject to recall error, the disability in the given ADL did not exist in the third month prior to interview. We assigned such months a nondisabled status.

We also assigned disability duration from detailed community interviews conservatively. For example, where a respondent indicated that a given ADL disability existed "6 months to less than 1 year," we assigned disability to the month of interview and the preceding 5 months. In instances where the duration assignment would have been prior to a previously reported functional status measure, we permitted that earlier information to override subsequently reported retrospective data, under the assumption that contemporaneous responses have more validity than later recollections.

The longest duration category was "5 years or over." Here we assigned a total of 60 months of disability, the month of interview and the 59 preceding months. Additionally in these instances of long-term disability, we examined the individual's status for the same ADL in the previous wave. Although by design the 1984–1989 and 1989–1994 intervals were 5 years, in practice we found a distribution of elapsed time between 2 successive interviews. When the individual had been disabled for "5 years or over" and had been disabled in the same ADL in the previous wave, we judged it was reasonable to assume that the long-term disability had lasted at least from the month of the preceding wave, and assigned disability in the array accordingly. In instances where the interval between interviews was less than 5 years, an analogous procedure was applied to duration intervals of "1 year to less than 5 years."

The final sample (*n *= 3440) excludes individuals who were removed from the 1989 survey frame due to funding limitations. We addressed this by re-weighting. The goal was to create a new weighted sample that would represent the full NLTCS, and therefore the older population of the United States. Using published procedures [20], we used logistic regression to estimate the probability that an individual would have each of a set of characteristics associated with being excluded. We then used this set of probabilities to adjust sample weights distributed with the NLTCS. We adjusted baseline NLTCS weights from 1982, because we were conducting a cohort analysis. We similarly reweighted a sub-sample of respondents who died before the 1989 survey. These decedents were over-represented in the final sample, compared with their representation in the full NLTCS. The goal of this reweighting was to ensure that decedents contributed the same proportions to the final sample and the full NLTCS.

We created two research data sets. One, which we call "sparse" data, includes one month of information from each survey wave in which a respondent participated. In each wave, this includes information from a detailed community or institutional interview, where available. For those institutionalized in 1989, we used the initial institutional interview, where available. In its absence, we used the institutional follow-up survey. In the absence of these sources, we used screening information. The month of death was also included, where known. Thus the sparse data could contain at most five months of information for each respondent, one for each survey wave plus the month of death. With the exception that our disability definition did not include "standby" help, the reweighted sparse data generally reproduced functional status prevalence estimates reported elsewhere based on the NLTCS. The second data set, which we call "dense" data, included the sparse data, plus additional information from screening for waves in which a respondent participated in both screening and detailed interviews, any information from both 1989 institutional interviews, plus all retrospective data described above. Most of the information added to the dense arrays reported disability among respondents represented as disabled in the sparse arrays. Such respondents represent a relatively small proportion of the overall sample, because most older persons at the ages we studied are not disabled. Within this group, however, the additions increase the available information substantially. Further details of the data and procedures are available [21].

### Estimating models of disability transitions

For both the sparse and the dense versions of the data, we estimated separate transition models for each of the six ADLs. This approach implicitly assumes that each ADL disability evolves independently of the other five. This is an unrealistic assumption. However, it was not our goal in this paper to model the joint dynamics of disability. Instead, our goal was to assess the impact of introducing the retrospective disability information. Likelihood ratio tests indicated that race-specific models were not justified in preference to pooled models with race categories distinguished using dummy variables. We therefore chose the latter approach.

For each of the six ADLs, we defined two transient functional status states, *nondisabled *and *disabled*. Death was included as an absorbing state. Thus we modeled a sequence of multichotomous variables *STATUS*_*t*_, which took values *N *(nondisabled), *D *(disabled), and *M *(dead). For each ADL, transitions between states were governed by 1-month transition probabilities, *p*_*jk*_(*X*) = *pr*(*STATUS*_*t*+1 _= *k *| *STATUS*_*t *_= *j*; *X*), where *X *was an array of explanatory variables that included *age *(in years) and race (a dummy variable indicating *nonwhite*). These probabilities were arranged in 3 × 3 Markovian transition matrices of the form

P(X)=[1−pND(X)−pNM(X)pND(X)pNM(X)pDN(X)1−pDN(X)−pDM(X)pDM(X)001]
 MathType@MTEF@5@5@+=feaafiart1ev1aaatCvAUfKttLearuWrP9MDH5MBPbIqV92AaeXatLxBI9gBaebbnrfifHhDYfgasaacH8akY=wiFfYdH8Gipec8Eeeu0xXdbba9frFj0=OqFfea0dXdd9vqai=hGuQ8kuc9pgc9s8qqaq=dirpe0xb9q8qiLsFr0=vr0=vr0dc8meaabaqaciaacaGaaeqabaqabeGadaaakeaacqWGqbaucqGGOaakcqWGybawcqGGPaqkcqGH9aqpdaWadaqaauaabeqadmaaaeaacqaIXaqmcqGHsislcqWGWbaCdaWgaaWcbaGaemOta4KaemiraqeabeaakiabcIcaOiabdIfayjabcMcaPiabgkHiTiabdchaWnaaBaaaleaacqWGobGtcqWGnbqtaeqaaOGaeiikaGIaemiwaGLaeiykaKcabaGaemiCaa3aaSbaaSqaaiabd6eaojabdseaebqabaGccqGGOaakcqWGybawcqGGPaqkaeaacqWGWbaCdaWgaaWcbaGaemOta4Kaemyta0eabeaakiabcIcaOiabdIfayjabcMcaPaqaaiabdchaWnaaBaaaleaacqWGebarcqWGobGtaeqaaOGaeiikaGIaemiwaGLaeiykaKcabaGaeGymaeJaeyOeI0IaemiCaa3aaSbaaSqaaiabdseaejabd6eaobqabaGccqGGOaakcqWGybawcqGGPaqkcqGHsislcqWGWbaCdaWgaaWcbaGaemiraqKaemyta0eabeaakiabcIcaOiabdIfayjabcMcaPaqaaiabdchaWnaaBaaaleaacqWGebarcqWGnbqtaeqaaOGaeiikaGIaemiwaGLaeiykaKcabaGaeGimaadabaGaeGimaadabaGaeGymaedaaaGaay5waiaaw2faaaaa@724F@

where the third row represented the probabilities *p*_*MN *_= *p*_*MD *_= 0 and *p*_*MM *_= 1, corresponding to the absorbing state, dead.

The first two rows of the *P*-matrix were parameterized as multinomial logistic regression models, whose off-diagonal entries are written as

ln⁡[pjk(X)pjj(X)]=Xβjk,
 MathType@MTEF@5@5@+=feaafiart1ev1aaatCvAUfKttLearuWrP9MDH5MBPbIqV92AaeXatLxBI9gBaebbnrfifHhDYfgasaacH8akY=wiFfYdH8Gipec8Eeeu0xXdbba9frFj0=OqFfea0dXdd9vqai=hGuQ8kuc9pgc9s8qqaq=dirpe0xb9q8qiLsFr0=vr0=vr0dc8meaabaqaciaacaGaaeqabaqabeGadaaakeaacyGGSbaBcqGGUbGBdaWadaqaamaalaaabaGaemiCaa3aaSbaaSqaaiabdQgaQjabdUgaRbqabaGccqGGOaakcqWGybawcqGGPaqkaeaacqWGWbaCdaWgaaWcbaGaemOAaOMaemOAaOgabeaakiabcIcaOiabdIfayjabcMcaPaaaaiaawUfacaGLDbaacqGH9aqpcqWGybawcqaHYoGydaWgaaWcbaGaemOAaOMaem4AaSgabeaakiabcYcaSaaa@47B0@

with *j *= *N *or *D*, *k *= *N*, *D*, or *M*, and *k *≠ *j*. Thus, logistic regression coefficients corresponding to the diagonal entries in *P *were normalized to zero.

The probability of making a transition from state *j *to state *k *over an interval of *w *months is the *j*, *k *entry of the matrix product *P*^*w*^, denoted *p*_*jk *_^(*w*)^, and the log-likelihood of the data, summed over intervals indexed by *n*, is

∑n=1Nln⁡pjnkn(w).
 MathType@MTEF@5@5@+=feaafiart1ev1aaatCvAUfKttLearuWrP9MDH5MBPbIqV92AaeXatLxBI9gBaebbnrfifHhDYfgasaacH8akY=wiFfYdH8Gipec8Eeeu0xXdbba9frFj0=OqFfea0dXdd9vqai=hGuQ8kuc9pgc9s8qqaq=dirpe0xb9q8qiLsFr0=vr0=vr0dc8meaabaqaciaacaGaaeqabaqabeGadaaakeaadaaeWbqaaiGbcYgaSjabc6gaUbWcbaGaemOBa4Maeyypa0JaeGymaedabaGaemOta4eaniabggHiLdGccqWGWbaCdaqhaaWcbaGaemOAaO2aaSbaaWqaaiabd6gaUbqabaWccqWGRbWAdaWgaaadbaGaemOBa4gabeaaaSqaaiabcIcaOiabdEha3jabcMcaPaaakiabc6caUaaa@41E1@

This approach accommodates intervals of any length between empirically observed functional statuses, permitting use of all observed intervals between interviews (and death) in the NLTCS. It also permits any unobserved pattern of transitions to intervene between each pair of known statuses. We obtained estimates of unknown parameters of the model through iterative maximization of the log-likelihood function, using a program written for this purpose in the SAS/IML™ language.

Our expectations concerning the impact of using the dense, in contrast to the sparse, data arrays reflect the fact that among persons coded as disabled, sequences of one or more missing monthly disability codes were replaced with the code for "disabled." Sequences of otherwise missing status codes were rarely replaced with the code corresponding to "nondisabled." The consequences of these changes for parameter estimates are ambiguous. When a sequence of missing values is replaced by a sequence of ones, indicating an unbroken series of months with disability, the estimated probability of *remaining *disabled should increase while the probability of recovery falls. However, adding that sequence of disability indicators also narrow the time intervals separating a month without disability from a later month of reported disability. This might tend to produce the opposite direction of change in the estimated probabilities of transition from nondisabled to nondisabled (lower) or to disabled (higher). Our primary hypothesis, therefore, is simply that the estimated probabilities of functional status change will be *different*, both statistically and substantively, when estimated using dense data instead of sparse data.

### Conducting microsimulations

To further illustrate the impact of adding retrospective information, we conducted microsimulations. Synthetic cohorts of 100,000 men and 100,000 women were created for each of the 6 ADLs. The baseline status of each cohort represented the race and disability distribution of its gender/ADL category for individuals aged 65–69 in the United States in 1984, as determined by weighted analysis of the 1984 NLTCS, including both community and institutional residents. The microsimulation procedure used the transition probability matrices to assign each simulated individual's survivorship and functional status for each month from the first of their 65^th ^year through 15 full years or their month of death, whichever came first. Thus the microsimulation was restricted to the age range observed in our data, and we do not report estimates of active life expectancy through death. Laditka and Wolf [11] provide further details of the procedure.

## Results

### Descriptive differences between sparse and dense data

Our coding procedures produced two different types of disability-status "episodes." One, "interval-censored" episodes, are defined by a month in which disability status is known, followed by one or more months in which it is unknown, followed by a final month in which it is again known. The other, "complete" episodes, consist of two or more successive months of known disability status that are both preceded by, and followed by, months in which disability status is unknown. Complete episodes can also end with the month of death. Often, a month in which a disability status is known will both end one episode and begin another.

Table 2 shows differences in interval-censored episodes between the sparse and dense data. Across ADLs, the dense data produce more of these episodes, with a grand mean increase across ADLs of 5.5%. As the number of such intervals increases, their mean length decreases by about 8%. The majority of older individuals at the ages represented in our data experienced few ADL disabilities, so the mean number and length of these episodes across data types for all observations do not change dramatically. Dense data impacts appear more clearly in a comparison of the median lengths of such episodes, where the average reduction from sparse to dense data across ADLs is 32.5%.

**Table 2:** Interval-Censored Episodes^a^

	Number of Episodes Per Observation					Episode Length					
		
	Mean (SD)		Min	Max		Mean (SD)		Median		Min	Max
	Sparse	Dense	Both	Sparse	Dense	Sparse	Dense	Sparse	Dense	Both	Both

ADL											
Eating	2.56 (0.72)	2.68 (0.93)	1	4	6	40.99 (19.73)	38.40 (21.20)	40	28	2	145
I/O Bed^b^	2.56 (0.72)	2.71 (0.87)	1	4	6	40.96 (19.70)	37.62 (21.35)	40	27	2	145
Mobility	2.57 (0.71)	2.73 (0.83)	1	4	6	41.02 (19.74)	37.26 (21.36)	40	27	2	145
Dressing	2.57 (0.71)	2.72 (0.89)	1	4	6	41.09 (19.79)	38.17 (21.33)	40	27	2	145
Bathing	2.57 (0.71)	2.69 (0.93)	1	4	6	41.08 (19.85)	37.19 (21.47)	40	26	2	145
Toileting	2.57 (0.71)	2.72 (0.89)	1	4	6	41.05 (19.77)	37.73 (21.33)	40	27	2	145

^a^Instances where a recorded functional status is followed by one or more months with no status information, followed by a non-missing functional status or month of death.

^b^I/O Bed = Getting in or out of bed.

Table 3 shows differences in "complete" episodes between the sparse and dense data. The first pair of columns shows the percentage of observations having one or more of these episodes. In sparse data, such sequences occur only in the few instances when a month of interview is followed immediately by the month of death. Only about 1% of observations have such sequences. However, across the ADLs in the dense data representation, about 25% of observations have one or more such sequences. The remaining columns of Table 2 are restricted to observations having such sequences. The mean number of sequences per observation is 1 in all sparse data ADLs (not shown). The grand mean of the number of such sequences per observation across ADLs is about 8.8% greater in the dense data. The sequence length is invariably 2 in sparse data, restricted to the few instances where a month of interview is followed immediately by death. In dense data, the grand mean of the sequence length across ADLs is 13.2 months. Also of particular interest in Table 3 are the maximum values for sequence lengths. That maximum is 2 in every instance for sparse data, as already noted. It is 190 months in the dense data mobility ADL, representing nearly 16 years of uninterrupted disability.

**Table 3:** Sequences of Non-missing Monthly Status^a^

	Observations with One or More Sequences		Number of Sequences per Observation			Length of Sequences						
			
	% of N^b^		Mean (SD)	Max		Mean (SD)		Median		Min	Max	
	Sparse^c^	Dense	Dense^d^	Sparse	Dense	Sparse^e^	Dense	Sparse	Dense	Both	Sparse	Dense

ADL												
Eating	1.16	19.45	1.044 (0.212)	1	3	2.00	4.79 (10.71)	2	2	2	2	133
I/O Bed^f^	1.16	25.87	1.091 (0.296)	1	3	2.00	13.86 (22.74)	2	2	2	2	164
Mobility	1.10	28.98	1.12 (0.339)	1	3	2.00	18.12 (27.85)	2	6	2	2	190
Dressing	1.08	23.72	1.071 (0.272)	1	3	2.00	11.34 (20.42)	2	2	2	2	133
Bathing	1.08	30.55	1.118 (0.339)	1	3	2.00	19.73 (27.98)	2	6	2	2	158
Toileting	1.13	24.01	1.083 (0.280)	1	3	2.00	11.86 (20.76)	2	2	2	2	141

^a^Monthly STATUS_t _followed by STATUS_t+1_, where STATUS is nonmissing, through month of death where available. Data columns 3–10 restricted to observations having such sequences. SD = Standard Deviation.

^b^N = 3440.

^c^Sparse = sparse data; Dense = dense data; See text for definitions. Sequences of non-missing monthly status information are available in sparse data only in instances when a month of interview is followed immediately by the month of death.

^d^Mean number of sequences in sparse data observations having a sequence is 1 in all instances.

^e^Standard deviation not applicable to sparse data sequence length.

^f^I/O Bed = Getting in or out of bed.

### Specification tests of nested estimators

We used the Hausman specification test [22,23] to determine if our estimates from sparse and dense were different. This test determines whether the difference between parameter sets estimated using an inefficient procedure, relative to those estimated using an efficient alternative, is statistically significant. In our application, the dense data produce maximum likelihood estimates that use all available information, and are therefore by definition efficient relative to the estimates based on the sparse data (which are a subset of the dense data). Table 4 reports results of these tests. For both women and men, in 4 of 6 ADLs the Hausman test indicates that the dense data estimates are significantly different from the sparse data estimates, at high levels of statistical significance. In general, then, we are led to reject the sparse-data models in favor of the dense-data models.

**Table 4:** Differences Between Estimations from Sparse and Dense Data, with Hausman Test Statistics (*q*)

	Men			Women		
		
	Sparse^a^	Dense	*q*	Sparse	Dense	*Q*
*ADL*						
Eating	3001.4	3140.2	0^c^	2910.1	3109.3	0^c^
I/O Bed^b^	3344.4	3842.6	553.4*	3519.1	4182.7	282.3*
Mobility	3441.1	4032.7	3098.1*	3771.7	4723.4	592.7*
Dressing	3244.2	3600.6	480.4*	3366.5	3885.4	0^c^
Bathing	3492.4	4088.9	0^c^	3947.2	4948.9	248.0*
Toileting	3298.6	3613.7	98.7*	3501.0	4109.2	362.1*

^a^Listed are -log likelihood values for estimates from sparse and dense data for each ADL.

^b^I/O Bed = Getting in or out of bed.

^c^Negative *q *values are treated as zeros [23,24].

*p < 0.0001

### Age profiles of transition probabilities

The individual coefficients of our models are difficult to interpret, as they represent partial effects in a multinomial-logit regression. However, for each combination of gender and race, and for each of 6 ADL measures, our model implies a transition probability matrix for each age beginning at 65. For each such matrix we can compare the individual probabilities obtained from the sparse and the dense data. At age 65, the probabilities for remaining nondisabled are slightly lower in all 24 sets of probabilities when estimated using dense data than they are when estimated using sparse data. All 24 of the probabilities of transitioning from disability to death are also lower at age 65 when estimated using dense data.

**Figure 1:**
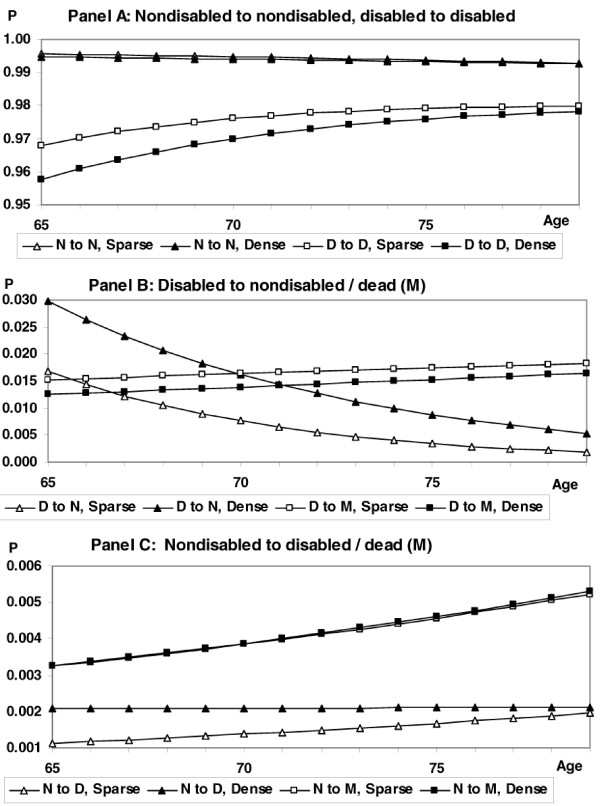
Transition Probabilities Estimated from Sparse and Dense Data, White Males, Mobility ADL, Ages 65–79; N = Nondisabled, D = Disabled, M = Dead; Data source: National Long Term Care Survey.

At age 65, all 24 probabilities for becoming disabled rise, and all 24 probabilities for remaining disabled fall, when dense data replace sparse data. The respective sets of probabilities estimated from sparse and dense data tend to converge by age 79, and a few reverse their relationships after age 75. Figure 1 illustrates both the effect of advancing age, and the effects of sparse and dense data, on the various transition probabilities. This illustration presents the mobility ADL for white men, which produced the largest Hausman test statistic (*q *= 3098.1), suggesting that the difference between the sparse and dense estimators was greatest for this ADL. Our discussion also describes common features of the age progression of probabilities across other gender/ADL combinations that are not shown in the figure.

As illustrated in Panel A of Figure 1, the probability of remaining nondisabled declines modestly with increasing age in linear fashion when calculated from either the sparse or dense data. In general, across the six ADLs for both men and women of both race classifications, the probabilities of remaining nondisabled across the age range calculated from dense data are slightly lower than those from sparse data. As is true for most other gender/race/ADL combinations, the probability of remaining disabled is lower at younger ages when calculated from dense data than it is when calculated from sparse data, although the tendency is for the respective probabilities to converge at later ages, or in some instances to cross after age 75. Thus, the rate at which the probability of remaining disabled rises with advancing age is greater when calculated from dense data than it is when calculated from sparse data. Note that, while the probabilities of recovery (Figure 1, Panel B) fall with advancing age (as is true for both women and men), these probabilities remain higher when estimated from dense data, although the difference between recovery probabilities estimated from sparse and dense data decreases throughout the age range. However, the rate at which the probability of recovering from disability declines with advancing age is greater when calculated from dense data than it is when calculated from sparse data. As for the transition from disability to death, Panel B of Figure 1 illustrates the common pattern for both women and men, where this probability is invariably lower for both whites and nonwhites across the six ADLs, and across the age range, when calculated from dense data. The findings for recovering, and for dying while disabled, suggest that recovering from disability may be more common than previously suspected. However, the combined effect of these findings and the result for remaining disabled is uncertain. These results also suggest that probabilities of dying while disabled calculated from sparse data are overestimated.

Across most race/ADL sets, two other consistent patterns are evident. The first is the pattern of relative probabilities for becoming disabled estimated from sparse and dense data across advancing ages (Panel C). With dense data, these probabilities are invariably higher at younger ages. With sparse data, these probabilities all rise throughout the age range. However, for most ADLs, the probabilities for becoming disabled estimated from dense data either remain relatively constant with advancing age, or decline. Thus, the sets of probabilities from sparse and dense data tend to converge at advanced ages. The second additional consistent pattern is the relative uniformity of the probabilities from sparse and dense data across ages for the transition from nondisabled to dead. Consistently, the probability of this transition is only slightly higher when computed using dense data. This is an expected result, because the dense data add only a small amount of information regarding months without disability, and no additional information regarding death.

### Microsimulation of functional status histories

Microsimulation can further illustrate effects of the various changes in the probability estimates just described. Results of microsimulations using transition probabilities estimated from sparse data ("sparse simulations") and from dense data ("dense simulations") are shown in Tables 5 (women) and 6 (men). The tables show the mean number of transitions from nondisabled to disabled or dead, and of transitions from disabled to nondisabled or dead, for each of six ADLs. Also shown are the means and standard deviations of sojourn-time distributions (in months). It should be noted that the standard deviations represent the dispersion of the random variable (duration) in the population. Thus, they are not affected by the size of the simulated population.

**Table 5:** Microsimulation Results: Sojourn Durations, Sparse vs. Dense Data, Simulated Populations of 100,000/ADL, Women

	Sparse Data				Dense Data^c^			
		
	Nondisabled to Disabled or Dead		Disabled to Nondisabled or Dead		Nondisabled to Disabled or Dead		Disabled to Nondisabled or Dead	
				
	mean n^a ^(SD)	mean duration^b ^(SD)	mean n (SD)	mean duration (SD)	mean n (SD)	mean duration (SD)	mean n (SD)	mean duration (SD)
ADL Eating	0.437 (0.509)	95.39 (50.62)	0.140 (0.356)	18.47 (19.01)	0.471 (0.529)	93.41 (50.48)	0.173 (0.398)	18.02 (18.22)
I/O Bed^d^	0.534 (0.552)	89.90 (50.39)	0.253 (0.470)	27.31 (26.34)	0.651 (0.618)	80.74 (49.60)	0.367 (0.564)	23.03 (22.63)
Mobility	0.556 (0.544)	87.72 (50.59)	0.262 (0.468)	37.47 (33.79)	0.696 (0.625)	77.47 (48.97)	0.400 (0.583)	27.48 (26.81)
Dressing	0.506 (0.639)	91.72 (50.60)	0.222 (0.439)	27.98 (26.50)	0.592 (0.592)	84.65 (50.45)	0.309 (0.518)	23.20 (22.53)
Bathing	0.602 (0.559)	86.07 (50.24)	0.302 (0.499)	37.00 (33.13)	0.722 (0.619)	77.65 (49.19)	0.418 (0.580)	32.79 (29.85)
Toileting	0.512 (0.531)	91.29 (50.81)	0.220 (0.436)	35.35 (32.61)	0.587 (0.572)	86.38 (50.05)	0.289 (0.497)	29.83 (27.82)

^a^Mean n calculated from full 100,000 simulated population; SD = Standard Deviation.

^b^Mean duration calculated as the mean of all existing transition intervals.

^c^Comparisons of population means between corresponding categories of sparse and dense data are significant at *p *< 0.0001 in all instances.

^d^I/O Bed = Getting in or out of bed.

**Table 6:** Microsimulation Results: Sojourn Durations, Sparse vs. Dense Data, Simulated Populations of 100,000/ADL, Men

	Sparse Data				Dense Data^c^			
		
	Nondisabled to Disabled or Dead		Disabled to Nondisabled or Dead		Nondisabled to Disabled or Dead		Disabled to Nondisabled or Dead	
				
	mean n^a ^(SD)	mean duration^b ^(SD)	mean n (SD)	mean duration (SD)	mean n (SD)	mean duration (SD)	mean n (SD)	mean duration (SD)
ADL Eating	0.586 (0.507)	84.95 (50.87)	0.147 (0.361)	18.37 (17.68)	0.678 (0.551)	80.41 (50.00)	0.221 (0.452)	9.33 (9.48)
I/O Bed^d^	0.636 (0.518)	82.72 (50.35)	0.196 (0.415)	25.07 (24.64)	0.709 (0.557)	78.23 (49.55)	0.267 (0.487)	21.43 (21.31)
Mobility	0.627 (0.512)	82.73 (50.38)	0.210 (0.421)	32.04 (30.11)	0.706 (0.547)	78.47 (49.67)	0.279 (0.490)	27.26 (26.08)
Dressing	0.615 (0.514)	82.99 (50.55)	0.179 (0.398)	24.27 (23.61)	0.682 (0.553)	79.34 (49.99)	0.245 (0.467)	18.85 (19.22)
Bathing	0.630 (0.515)	81.80 (50.58)	0.239 (0.443)	31.59 (29.21)	0.731 (0.564)	75.31 (49.37)	0.336 (0.527)	24.12 (31.40)
Toileting	0.613 (0.510)	83.57 (50.76)	0.188 (0.402)	28.85 (26.85)	0.662 (0.534)	79.96 (49.96)	0.229 (0.447)	23.75 (23.56)

^a^Mean n calculated from full 100,000 simulated population; SD = Standard Deviation.

^b^Mean duration calculated as the mean of all existing transition intervals.

^c^Comparisons of population means between corresponding categories of sparse and dense data are significant at *p *< 0.0001 in all instances.

^d^I/O Bed = Getting in or out of bed.

For women, the mean number of transitions from nondisabled to either disabled or dead is 0.556 in the sparse mobility simulation, for example. The corresponding mean from the dense simulation is 0.696, a 25.2% increase. The mean number of such transitions rises from sparse to dense simulations for both women and men in all ADLs, with a grand mean rise of 17.7% for women, 12.5% for men. As the number of transitions increases, we would expect the mean sojourn-time durations to decrease. This is the pattern for both women and men, with 7.8% and 5.4% grand mean duration reductions, respectively, across the six ADLs. As with the transition probabilities that govern the simulation, this transition and its sojourn length have the expected direction of change, given the respective additions to dense data of information about individuals being disabled or nondisabled. The same pattern is found when examining spells that begin with disability. In this instance, however, the magnitude of change from sparse to dense simulations is greater. The grand mean number of transitions from disabled to either nondisabled or dead increases by 38.4% for women, 36.4% for men. The grand mean of the sojourn-time duration decrease is 14.8% for women, 23.7% for men.

For women, the mean number of transitions from disabled to either nondisabled or dead is 0.262 in the sparse mobility simulation, for example. The corresponding mean from the dense simulation is 0.400, a 52.7% increase. The mean number of such transitions increases from sparse to dense simulations for both women and men in all ADLs, with a grand mean increase of 38.4% for women, 36.4% for men.

## Discussion

We examined the hypothesis that adding retrospective disability information to analyses using panel survey data would improve estimates of disability processes. Our analysis supported the hypothesis. Estimates of probabilities predicting functional status transitions were significantly changed by retrospective disability information. The differences between estimated transition probabilities derived from sparse and dense data were substantial. Results of simulations based on these probabilities also differed substantially. These results suggest that dense data estimates will alter our understanding of functional status dynamics. When investigators use the "sparse" data that are available in most panel surveys to estimate disability processes, it is likely that:

• The probability of recovering from disability is underestimated;

• The probability of dying while disabled is overestimated;

• For those who are not disabled, the probability of becoming disabled or dying is underestimated;

• The number of functional status transitions is underestimated; and

• The length of time a typical individual spends in a given episode of being either disabled or nondisabled is overestimated.

These over- and under-estimations associated with using sparse data are of considerable magnitude. Our results suggest that surveys could better capture disability dynamics even where funding limitations and concerns about respondent burden prohibit more frequent survey waves. "Telescoping" techniques, in which respondents are asked, "since we last interviewed you in (month, year), have you had any difficulty in [ADL]," possibly followed by questions on dates of onset and recovery, could provide more information about processes of disability and recovery.

We noted at the outset a key limitation of the partial retrospective data used in our analyses. While retrospective information was available in our data on the length of spells-in-progress among the disabled, there was no parallel information on the length of spells-in-progress among the *non*disabled. To address this limitation, analogous questions could be asked of those reporting no current disability: "Have you experienced *any *months of disability in [ADL] since we last interviewed you in (month, year)?" In the dense data of our analysis, a negative response to this question would allow retrospective assignment of nondisabled status throughout the entire period between the previous and current waves. Those who had already indicated no disability in the given ADL at the time of the current interview, but who respond affirmatively to this question, might be asked about the onset and duration of the past disability, or the amount of time since recovery. Despite the limitations of recall, these additions would likely improve on the functional status histories widely used heretofore, where known statuses at end-points of intervals lasting several years have often been presumed to indicate a lack of intervening transitions.

Another limitation of our analysis is that we develop models for each of six ADL measures, as though each disability indicator evolves independently of the others. In contrast, the preferred approach generally found in the literature represents ADL disability using a scale, for example a count (from 0 to 6) of the number of tasks in which subjects are limited or dependent. Our approach is, however, dictated by NLTCS question wording and data-coding procedures. For example, a respondent may at the time of interview be disabled in one ADL, and report that they have experienced this disability for "1 year to less than 5 years." If they are at the time of the interview nondisabled in the other 5 ADLs, then their disability status for the other ADLs is coded "missing" for the past 5 years. For such a person, it is not possible to determine the value of a summary ADL index in any month prior to the interview month.

Finally, our approach requires that we mix contemporaneous with retrospective self-reports of disability status. Measurement error must be suspected in any self-reported disability measure; the issue for our analysis is whether there is *differential *measurement error in the two types of survey responses. One reason for optimism regarding the quality of the NLTCS retrospective data is that questions about the duration of disability come immediately after positive responses to questions about the contemporaneous presence of disability. Thus, any errors present in the contemporaneous reports will at least tend to be consistent with the errors present in the retrospective reports. A small existing literature investigates the quality of retrospective reports of disability. One study of retrospective reports of ADL functioning, collected at the time of hospital admission, concluded that the retrospective data had face validity as well as predictive validity, and also high prognostic value [25]. Unfortunately, the period of retrospection used in that study went back only two weeks. Another study suggested that subjects currently experiencing an adverse condition or symptom (specifically, depression) provide more accurate retrospective data (about the condition) than do others [26]. This finding is particularly relevant to our research, in which retrospective data are collected only from those reporting the presence of disability.

## Conclusion

Our results suggest that the value of future panel surveys for analyzing disability processes could be notably enhanced by adding a small number of questions asking respondents for details about periods with and without disability in the time since a preceding survey wave. The addition of such questions would add modestly to respondent burden and measurement error. This addition also involves a design challenge, as retrospective questions cannot be asked of those who have died, or who for some other reason become unavailable to follow-up. Our results suggest that the information provided by such questions could nonetheless substantially enhance the measurement of disability histories. This enhancement holds the potential to improve estimates of disability processes.

## Abbreviations

ADL Activity of Daily Living

EMC Embedded Markov Chain

## Competing interests

The author(s) declare that they have no competing interests.

## Authors' contributions

JNL participated in the design of the analysis, prepared the data for analysis, conducted the descriptive and statistical analyses, and prepared the tables and figures. DAW designed the study, developed the statistical models, and participated in the analysis. Both authors participated in drafting the manuscript. Both authors read and approved the final manuscript.
